# Smoking cessation after cancer diagnosis reduces the risk of severe cancer pain: A longitudinal cohort study

**DOI:** 10.1371/journal.pone.0272779

**Published:** 2022-08-09

**Authors:** Chie Taniguchi, Akihiko Narisada, Hideo Tanaka, Hiroki Iida, Mami Iida, Rina Mori, Ayako Nakayama, Kohta Suzuki

**Affiliations:** 1 College of Nursing, Aichi Medical University, Nagakute, Aichi, Japan; 2 Institute for Occupational Health Science, Aichi Medical University, Nagakute, Aichi, Japan; 3 Public Health Center of Neyagawa City, Neyagawa, Osaka, Japan; 4 Shiga University of Medical Science, Otsu, Shiga, Japan; 5 Department of Anesthesiology and Pain Medicine, Central Japan International Medical Center, Minokamo, Gifu, Japan; 6 Department of Internal Medicine and Cardiology, Gifu Prefectural General Medical Center, Gifu, Gifu, Japan; 7 Department of Health and Psychosocial Medicine, Aichi Medical University School of Medicine, Nagakute, Aichi, Japan; University of Tokyo, JAPAN

## Abstract

**Background:**

Whether abstinence from smoking among cancer patients reduces cancer pain is still unclear. Opioids can act as a surrogate index for evaluating the incidence of severe cancer pain in countries where opioid abuse is infrequent. This study aimed to investigate whether changed smoking behavior after cancer diagnosis influences the incidence of severe cancer pain as determined by strong opioid use.

**Methods:**

Using a large Japanese insurance claims database (n = 4,797,329), we selected 794,702 insured employees whose annual health checkup data could be confirmed ≥6 times between January 2009 and December 2018. We selected 591 study subjects from 3,256 employees who were diagnosed with cancer pain and had health checkup data at the year of cancer pain diagnosis.

**Results:**

A significantly greater proportion of patients who continued smoking after cancer diagnosis (“current smoker”, n = 133) received strong opioids (36.8%) compared with patients who had never smoked or had stopped before cancer diagnosis (“non-smoker”, n = 383, 20.6%; p<0.05) but also compared with patients who had quit smoking after cancer diagnosis (“abstainer:”, n = 75, 24.0%; p<0.05). In multivariable Cox proportional hazards regression analysis, abstainers had a significantly lower risk of receiving strong opioids than current smokers (hazard ratio: 0.57, 95% CI: 0.328 to 0.997). These findings were consistent across multiple sensitivity analyses.

**Conclusion:**

Our study demonstrated that patients who quit smoking after cancer diagnosis have a lower risk of severe cancer pain. This information adds clinical incentives for improving quality of life among those who smoked at the time of cancer diagnosis.

## Introduction

Cancer pain reduces the quality of life of cancer patients. Its prevalence is distressingly as high as 30–40% in patients during treatment, rising to 60–90% in patients with advanced disease [1]. Thus, the World Health Organization (WHO) has developed guidelines for the use of drugs to manage cancer pain and recommends a three-step pain ladder [2]. This suggests starting with weaker drugs, and then climbing the ladder if pain is still present at each step. When pain occurs, the first step is to use non-opioid drugs such as acetaminophen or NSAIDs. If complete pain relief is not achieved, the second step is to use weak opioids such as codeine or tramadol, added to the first step. If this becomes insufficient, the third step uses a strong opioid, such as morphine, fentanyl, oxycodone, or hydromorphone.

Some experimental studies suggest that nicotine has analgesic properties, probably exerted through the effect of central and peripheral nicotine acetylcholine receptors [3, 4]. This analgesic effect of nicotine would be more likely to reinforce individual smoking behavior. Long-term exposure to nicotine induces tolerance due to desensitization of nicotine acetylcholine receptors, which increases the amount of cigarettes smoked. Patients using smoking as a means to manage their pain reinforce their high nicotine dependence [5]. Therefore, smokers have been reported to be at an increased risk of chronic pain [6]. Additionally, over the last decade, reports that smoking is related to cancer pain have emerged [7] indicating that current smokers have higher levels of cancer pain than non-smokers [7–9]. However, these studies were cross-sectional [7–9] or cohort studies in which smoking status was recorded at the time of receiving supportive care [10] or evaluating the intensity of cancer pain simultaneously with recording smoking status [11]. Thus, causality in the relationship between smoking and cancer pain remains unclear.

There are two important points to clarify regarding associations between smoking and cancer pain. First, longitudinal studies based on an appropriate design are essential, such as prospective cohort studies, but to best of our knowledge, there have been no such investigations assessing the effect on pain of smoking cessation after cancer diagnosis. Second, it is necessary to objectively evaluate the degree of cancer pain. Some previous studies directly evaluated pain intensity using the Brief Pain Inventory (BPI) [9, 11, 12] and Numeric Rating Scale (NRS) [7]. Because pain is a subjective sensation, it is experienced very differently by different patients. It is also difficult to assess pain levels at different time points, especially in a prospective study. Therefore, to evaluate the worsening of cancer pain, it can be appropriate to apply the surrogate of analyzing opioid prescribing. Although some previous retrospective studies were performed to assess the association between intensity of cancer pain and smoking status using prescribed opioid doses, the accuracy of pain evaluation in those studies might have been confounded by the inclusion of persons with opioid use disorders [12, 13]. Opioids can act as an index for evaluating the intensity of cancer pain only in countries where opioid abuse is very infrequent [14]. This is the case in Japan [15] where opioid therapy has been regulated strictly for many years. Unlike in most developed countries, there is a legal requirement in Japan for every physician who administers narcotics to be licensed by the prefectural governor. In addition, health insurance companies cover oxycodone only when prescribed for cancer pain. For the above reasons, the prevalence of opioid abuse in Japan affected only 0.01% of the population and only six related deaths were reported in 2015 [15]. Therefore, a cohort study conducted in the Japanese population is considered to be suitable for assessing causal relationships between smoking status and cancer pain by leveraging opioid use as a surrogate marker.

Importantly for this study, Japan has universal medical insurance enabling everyone to access appropriate services. Medical claims databases including information on individual diagnoses, prescriptions and smoking status for the general population can be accessed from the medical insurance system. Thus, we aimed to investigate whether changing smoking behavior after cancer diagnosis influences the incidence of severe cancer pain by using analyzing a medical claims dataset in Japan.

## Materials and methods

### Study design and data sources

This study was a real-world, longitudinal cohort study using a large claims database constructed by the Japan Medical Data Center Co., Ltd (JMDC, Tokyo, Japan). This covers >7.3 million employees and their family members aged 40–74 years (approximately 3.2% of the Japanese population) since January 2005. In Japan, employers must provide for annual health checkups of employees conducted by a physician according to the stipulations of the Industrial Safety and Health Act. This database records information on the insured persons, including age, sex, date of birth, medical institution data (inpatient, outpatient, pharmacy), and annual health checkup data (anthropometric measurements, laboratory test results, and lifestyle behaviors: smoking status, regular exercise, sleep habits, etc.). Information in the context of the International Classification of Diseases 10th revision (ICD-10) status is contained in this database (i.e. diagnostic codes as well as the name of the drug provided and the number of days supplied on prescription). In addition, the Anatomical Therapeutic Chemical classifications of the European Pharmaceutical Market Research Association (EPHMRA) were used for drug coding. We used a medical claims database, so patients were not involved in this study, only their anonymous data. This study was approved by the Institutional Review Board of Aichi Medical University (ID: 2020–082).

### Study population

We identified 794,702 individuals where health checkup data could be confirmed at least 6 different time points between January 2009 and December 2018 from a total population of 4,797,329 insured persons. We extracted the records of 127,050 patients who were diagnosed or suspected cases of “cancer” (ICD-10 code C00-D48) without lymphoma, hematologic cancer, or benign tumors (ICD-10 codes C 81–96 and D 10–36). Of these, we selected 3,221 employees who had a diagnosis of “cancer pain” (ICD-10 code R522). Those who did not have information on smoking status for the year of cancer diagnosis and cancer pain diagnosis were excluded. In addition, we excluded cases of “those whose date of cancer pain diagnosis was identical to the first day of strong opioid prescription”, “suspected cancer pain”, and “those who started smoking after cancer diagnosis”. Finally, 591 patients remained for this study.

### Study outcome and definition

The primary study endpoint was establishing associations between strong opioid use and smoking status. The starting date for each patient was defined as the date of diagnosis of cancer pain. The end of the follow-up was defined as the first date on which strong opioids were prescribed, the date of death, date of last treatment in hospital, and 1000 days after the starting date or the closing date of the study, December 31, 2018, whichever occurred first.

Definition of strong opioid used the concept of the “analgesic ladder” created by the WHO as guidelines for using medication for pain management [16]. We defined the following six types of opioid as strong: oxycodone, hydromorphone, fentanyl, methadone, morphine, and tapentadol. Only oral and patch-type formulations were included.

Information on smoking status was extracted from the annual health checkup data as “smoking” or “not smoking” based on self-administered questionnaires. If both at the year of cancer diagnosis and cancer pain diagnosis the status was “not smoking”, the patients were classified as “non-smokers”. If the smoking status at the year of cancer diagnosis was “smoking” and subsequently at the year of cancer pain diagnosis was “not smoking”, patients were classified as “abstainers”. If patients were smoking at the time of both diagnoses, they were classified as “current smokers”.

### Primary analysis

The Chi-square test and Man-Whitney U test were used to compare the frequency of the baseline characteristics age (<60 years/≥60 years), sex, alcohol consumption (none, sometimes, every day), body mass index (BMI) at the time of cancer pain (<18.5 ≥18.5 < 25 ≥25), cancer type (not tobacco-related cancer/tobacco-related cancer: lung, head and neck (except thyroid), esophageal, pancreas, kidney, urinary bladder, renal pelvis, stomach, liver, uterine cervix cancer) [17], time period between cancer diagnosis and cancer pain (months), according to smoking status. Also, we calculated the proportion of patients using strong opioids according to type of cancer.

The Kaplan-Meier method was used to calculate the cumulative rate of continuing cancer treatment without requiring treatment with strong opioids, and the log-rank test was used to compare the likelihood of strong opioid use by non-smokers, abstainers, or current smokers. To identify relationships between the risk of strong opioid use and smoking status, we performed Cox proportional hazards regression analysis using a four-step modeling procedure (not adjusted, model A, B, and C), by increasing the number of covariates at each. In the first step, univariate Cox proportional hazards regression analysis was performed. In the second step (Model A), we added age and sex as covariates; Model B then also included alcohol consumption and BMI at the time of cancer pain diagnosis using Model A variables. The last step (Model C) added cancer type and duration between cancer diagnosis and cancer pain diagnosis to Model B variables. Finally, to quantify decreased risk of strong opioid use in abstainers, we performed Cox proportional hazards regression analysis with current smokers as the reference adjusted for the same covariates in Model C.

Data analysis was performed with STATA ver.16 software (STATA Corp, College Station, TX). A *P*-value <0.05 was considered statistically significant.

### Sensitivity analyses

Because we extracted information on smoking from annual health checkup data, this study possibly included misclassification of the smoking status at cancer pain diagnosis (S1 Fig). Therefore, we performed sensitivity analysis in three patterns to assess the robustness of the study findings as follows: Pattern 1: Patients with <1 year from cancer diagnosis to cancer pain diagnosis and whose smoking status was “not smoking” were judged to be abstainers, because those classified as non-smokers under these conditions might have included some abstainers. Pattern 2: Current smokers with <1 year from cancer diagnosis to cancer pain diagnosis who received health check-ups before cancer diagnosis were excluded, because this group could possibly include abstainers. Pattern 3: All patients with <1 year between cancer diagnosis and cancer pain diagnosis were excluded, because of the possibility of misclassification between non-smokers and abstainers.

## Results

### Characteristics of the study subjects

A total of 591 cancer pain patients was recruited of whom 383 (64.8%) were non-smokers according to the definition given in Methods above, 75 (12.7%) were abstainers, and 133 (22.5%) were current smokers. The demographic characteristics of these three groups are summarized in Table 1. The majority (80.7%) of patients was <60 years of age, and 72.4% were male. Of these 591 patients, 289 (48.9%) were diagnosed with tobacco-related cancers, comprising lung, head and neck, esophageal, pancreas, kidney, urinary bladder, renal pelvis, stomach, liver, and uterine cervix cancer. The mean duration between cancer diagnosis and cancer pain diagnosis was 25.8 months. Current smokers included a higher proportion of men, and a higher proportion of those who drank alcohol every day than the non-smokers. On the other hand, abstainers also had a higher proportion of men, daily drinkers, a higher proportion of tobacco-related cancer, and a longer time between cancer diagnosis and cancer pain diagnosis than non-smokers.

**Table 1:** Characteristics of the study subjects (n = 591).

		Non-smoker		Abstainer			Current smoker			Total	
		(n = 383)		(n = 75)			(n = 133)			(n = 591)	
		n	%	n	%	p-value	n	%	p-value	n	%
Age	<60 y	309	80.7	61	81.3	0.895	107	80.5	0.954	477	80.7
	≥60 y	74	19.3	14	18.7		26	19.6		114	19.3
Gender	Female	142	37.1	10	13.3	<0.001	11	8.3	<0.001	163	27.6
	Male	241	62.9	65	86.7		122	91.7		428	72.4
Alcohol consumption	None/sometimes	317	82.8	60	80.0	0.566	82	61.6	<0.001	459	77.7
	Every day	66	17.2	15	20.0		51	38.4		132	22.3
BMI at the time of cancer pain	<18.5	33	8.6	9	12.0	0.522	15	11.3	0.394	57	9.6
	≥18.5 < 25	275	71.8	46	61.3		83	62.4		404	68.4
	≥25	75	19.6	20	26.7		35	26.3		130	22.0
Cancer type ^a^	Not tobacco-related cancer	216	56.4	33	44.0	0.049	53	39.9	0.001	302	51.1
	Tobacco-related cancer	167	43.6	42	56.0		80	60.1		289	48.9
Duration ^b^	mean (SD)	24.1	(26.3)	39.1	(29.6)	<0.001	23.1	(26.3)	0.441	25.8	(27.2)

^a^: Tobacco-related cancer: lung, head and neck (except thyroid), esophageal, pancreas, kidney, urinary bladder, renal pelvis, stomach, liver, uterine cervix

^b^: Time between cancer diagnosis and occurrence of cancer pain (months)

Two-group comparison with non-smokers (Chi-square test / Man-Whitney U test)

More than 30% of strong opioid users were patients with head and neck cancer (38.6%), or stomach cancer (35.1%), both smoking-related (Table 2).

**Table 2:** Type of cancer diagnosis (n = 591).

	Type of cancer	n	Strong opioid use n	%
Tobacco-related cancer	Lung	84	21	25.0
	Head and Neck	44	17	38.6
	Esophagus	8	1	12.5
	Pancreas	34	7	20.6
	Kidney/Urinary bladder/Renal pelvis	31	6	19.4
	Stomach	57	20	35.1
	Liver	25	6	24.0
	Uterine cervix	6	1	16.7
Total		289	79	27.3
Not tobacco-related cancer	Colon	92	26	28.3
	Prostate	31	8	25.8
	Breast	47	3	6.4
	Bone	14	2	14.3
	Others	118	28	23.7
Total		302	67	22.2

### Kaplan-Meier analysis of the likelihood of strong opioid use

**Fig 1:**
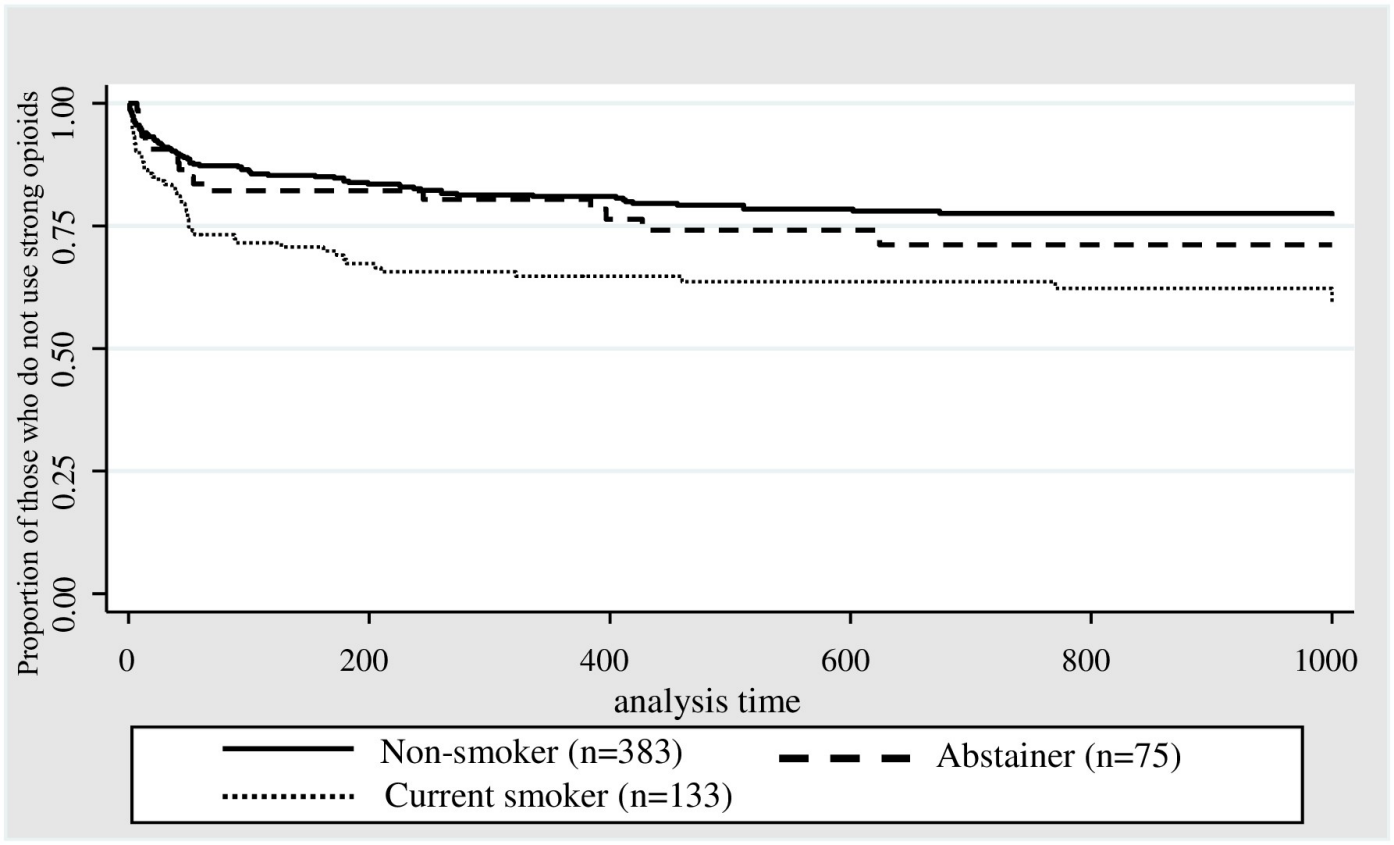
Kaplan–Meier analysis of the likelihood of strong opioid use according to smoking status (n = 591). Log-rank test: Non-smoker group/current smoker group: p<0.001. Non-smoker group/abstainer group: p = 0.401. Abstainer group/current smoker group: p<0.001.

There were 93 censored cases (74 deceased, and 19 dropped out). A total of 146 patients (24.7%) used strong opioids over a mean follow-up of 25.8 months. The likelihood of strong opioid use is shown in Fig 1 for the non-smoker group (n = 383; cumulative risk of strong opioid use: 20.6% (n = 79)), versus abstainers (n = 75; 24.0% (n = 18)), and current smokers (n = 133; 36.8% (n = 49)). These differences were significant between non-smokers and current smokers (log-rank test, p<0.001) and between abstainers and current smokers (log-rank test, p <0.001). In contrast, the difference between abstainers and non-smokers was not significant (p = 0.401).

### Risk of strong opioid use according to smoking status

Univariate Cox proportional hazards regression analysis was performed considering smoking status and possible confounding factors including age, gender, alcohol consumption, BMI, cancer type, duration between cancer diagnosis and cancer pain occurrence (Table 3). Current smokers had a significantly higher likelihood of strong opioid use (hazard ratio (HR):2.01; (95% confidence interval (CI): 1.41–2.88), whereas abstainers had an insignificant risk (HR:1.24; 95% CI: 0.85–2.08). We next performed multivariable Cox proportional hazards regression analysis increasing the number of covariates in three models to assess relationships between smoking status and strong opioid use. Current smokers were more likely to be using strong opioids than non-smokers even when adjusting for other possible factors (Models A, B, C yielded HRs of 1.67 (95% CI: 1.16–2.40), 1.71 (1.18–2.49), and 1.77 (1.21–2.58), respectively (Table 3). The HR for abstainers remained statistically insignificant (HR range 0.91–1.08, Table 3). With current smokers as the reference population in Cox proportional hazards regression analysis, abstainers had a significantly lower risk of strong opioid use (HR: 0.56, 95% CI: 0.32–0.97, Table 4).

**Table 3:** Factors associated with strong opioid use in patients diagnosed with cancer pain.

		Not adjusted			Model A			Model B			Model C		
		HR	p-value	[95% CI]	HR	p-value	[95% CI]	HR	p-value	[95% CI]	HR	p-value	[95% CI]
Smoking status	Non-smoker	ref			ref			ref			ref		
	Abstainer	1.24	0.403	[0.85–2.08]	1.07	0.793	[0.64–1.79]	1.08	0.765	[0.64–1.82]	0.91	0.719	[0.53–1.54]
	Current smoker	2.01	<0.001	[1.41–2.88]	1.67	0.006	[1.16–2.40]	1.71	0.005	[1.18–2.49]	1.77	0.003	[1.21–2.58]
Age	<60	ref			ref			ref			ref		
	≥60	0.83	0.425	[0.53–1.30]	0.76	0.228	[0.48–1.19]	0.77	0.268	[0.49–1.22]	0.70	0.127	[0.44–1.11]
Gender	Female	ref			ref			ref			ref		
	Male	2.14	<0.001	[1.55–2.95]	2.05	<0.001	[1.46–2.88]	2.14	<0.001	[1.51–3.01]	2.13	<0.001	[1.50–3.02]
Alcohol consumption	none/sometimes	ref						ref			ref		
	Every day	1.14	0.514	[0.78–1.65]				0.82	0.336	[0.56–1.22]	0.88	0.538	[0.60–1.31]
BMI at the time of cancer pain	<18.5	ref						ref			ref		
	≥18.5 < 25	0.80	0.381	[0.48–1.32]				0.75	0.273	[0.45–1.25]	0.74	0.240	[0.44–1.23]
	≥25	0.64	0.145	[0.35–1.17]				0.58	0.081	[0.32–1.07]	0.55	0.053	[0.30–1.01]
Cancer type ^a^	Not tobacco-related cancer	ref									ref		
	Tobacco-related cancer	1.38	0.056	[0.99–1.91]							1.09	0.619	[0.78–1.53]
Duration ^b^	(continuous variable)	1.01	0.002	[1.003–1.015]							1.01	0.001	[1.005–1.017]

^a^: Tobacco-related cancer: lung, head and neck (except thyroid), esophageal, pancreas, kidney, urinary bladder, renal pelvis, stomach, liver, uterine cervix

^b^: Time between cancer diagnosis and cancer pain (months)

Covariate: Model A: age and sex; Model B: age, sex, alcohol consumption and BMI; Model C: age, sex, alcohol consumption, BMI cancer type and duration between cancer diagnosis and cancer pain diagnosis.

**Table 4:** HR for strong opioid use taking current smokers as the reference.

	HR	p-value	[95% CI]
Current smoker	ref		
Abstainer	0.56	0.039	[0.32–0.97]
Non-smoker	0.63	0.017	[0.44–0.92]
Pattern 1			
Current smoker	ref		
Abstainer	0.61	0.014	[0.41–0.90]
Non-smoker	0.58	0.018	[0.37–0.91]
Pattern 2			
Current smoker	ref		
Abstainer	0.56	0.053	[0.31–1.01]
Non-smoker	0.63	0.032	[0.41–0.96]
Pattern 3			
Current smoker	ref		
Abstainer	0.43	0.014	[0.22–0.84]
Non-smoker	0.51	0.007	[0.31–0.83]

Adjusted for age (<60 year/≥60 year), sex, alcohol consumption (none, sometimes/every day), body mass index (BMI) at the time of cancer pain (<18.5 / ≥18.5 ≤ 25 / >25), cancer type (not tobacco-related cancer/tobacco-related cancer: lung, head and neck (except thyroid), esophageal, pancreas, kidney, urinary bladder, renal pelvis, stomach, liver, uterine cervix cancer), duration between cancer diagnosis and cancer pain (months).

### Sensitivity analysis

In multiple sensitivity analyses, changing the assessment of smoking status did not appreciably change the Kaplan-Meier estimates (S2 Fig). Further, findings with non-smokers as the reference population were consistent with three patterns (HRs for current smokers were as follows: Pattern 1: 1.94 (95% CI: 1.24–3.03), Pattern 2: 1.59 (95% CI: 1.04–2.43), Pattern 3: 2.41 (95% CI: 1.48–3.93) (S1 Table), whereas HRs for the abstainers were Pattern 1: 1.09 (95% CI: 0.70–1.69), Pattern 2: 0.89 (95% CI: 0.53–1.52), Pattern 3: 0.88 (95% CI: 0.47–1.66)) (S1 Table). With current smokers as the reference population, the HR for abstainers still indicated a lower risk of strong opioid use as shown in Table 4 (Pattern 1: HR 0.61 (95% CI: 0.41–0.90), Pattern 2: 0.56 (95% CI: 0.31–1.01), Pattern 3: 0.43 (95% CI: 0.22–0.84).

## Discussion

Our analysis of smoking status and likelihood of strong opioid use in Japanese cancer patients revealed that patients who continued to smoke after cancer diagnosis had a higher risk of strong opioid use than non-smokers. In addition, patients who quit smoking after cancer diagnosis were less likely to use strong opioids than those who continued to smoke. These findings were consistent across multiple sensitivity analyses.

In order to evaluate whether smoking status including abstainers influences the intensity of cancer pain using the surrogate of assessing strong opioid use, it is essential that the study cohort does not include persons with opioid use disorders at baseline. In addition, smoking status needs to be documented before the occurrence of cancer pain. Previous studies investigating associations between smoking status and cancer pain assessed by prescribed opioid use have not reported any causal relationships, but may have included persons with opioid use disorders in the study subjects [10, 12, 13, 18]. Under these circumstances, opioid use is an inappropriate surrogate pain indicator [10, 12, 13, 18]. Also, to unequivocally clarify the situation, large datasets are needed to follow-up patients with cancer pain. The present study provides robust real-world evidence from a large administrative claims database of opioid use in patients with cancer in Japan. Cancer patients who were targeted in this study were Japanese company employees at the year of cancer pain determination, receiving annual health checkups according to the requirements of the Industrial Safety and Health Act. Therefore, our study subjects are likely to have a lower incidence of cancer pain than the general cancer population. Furthermore, our study outcome is defined as strong opioid only orally or patch, not injection. If the study subjects include end-of-life stage patients, the association between opioid use and smoking cannot be evaluated accurately because opioid use increases rapidly before cancer death.

Here, we found that patients who continued to smoke after cancer diagnosis had a higher risk of strong opioid use than non-smokers. This finding indicates that continuing to smoke after cancer diagnosis is associated with severe cancer pain. Previous studies suggested that smokers did suffer higher levels of pain than non-smokers [19–21]. Nicotine activates the endogenous opioid system and rapidly develops analgesic properties on short term exposure [4, 22, 23]. However, long-term nicotine exposure results in tolerance to nicotine-induced antinociception [5]. In addition, nicotine withdrawal is reported to enhance the perception of pain in experimental animals [24, 25]. The effects of smoking on pain could reinforce smoking behavior by the transient pain relief. Chronic exposure to nicotine increases pain sensitivity and causes hyperalgesia [26]. Similar mechanism could explain why cancer patients who continued to smoke have more severe pain.

In contrast, the probability of strong opioid use by patients with cancer pain who quit smoking after the cancer diagnosis was no different from non-smokers. Moreover, they had lower risk of strong opioid use than continuous smokers. This suggests that smoking cessation after a cancer diagnosis lowers the risk of subsequent strong cancer pain. Both nicotine and opioids act on the reward system with complex nicotine-opioid interactions [27]. Nicotine reinforcement partly depends on the opioid system, and the nicotine-acetylcholine system regulates opioid reinforcement [27]. In the short term, as systemic nicotine mediates acute analgesia, abstinence could exacerbate painful symptoms and also disallow a strategy seen by many smokers as helpful for controlling stress and anxiety. Nicotine withdrawal symptoms accompanying sudden abstinence might also complicate efforts to treat the pain [20]. Considering these mechanisms, the effects of analgesic opioid use are possibly manifested after abstinence is stabilized.

There is convincing evidence that preoperative smoking cessation can reduce postoperative complications in lung [28], head and neck [29] and gastric cancer [30] patients. Some cohort studies indicate that cancer patients who quit smoking at the time of cancer diagnosis survive longer on chemo- [31] and radiation [32] therapy. Despite these beneficial effects of smoking cessation in cancer patients, approximately 50% of current smokers who have cancer continue to smoke after the diagnosis [33]. Our finding that smoking cessation after cancer diagnosis can lower the likelihood of severe cancer pain adds clinical incentives to discouraging smoking even after cancer diagnosis.

This study had some limitations that must be considered. The first limitation is that this study possibly included misclassification of smoking status at the time of cancer pain diagnosis, because we extracted smoking information from annual health checkup data. Therefore, to mitigate against this, we performed sensitivity analyses of three patterns to assess the robustness of the study findings. Second, since the subjects of this study were cancer patients who were able to work even after the cancer diagnosis, it is considered that their physical condition was better and the incidence of cancer pain was lower than the general cancer patients. However, as we analyzed the relationship between smoking status and strong opioid use in the same settings, this relationship would not be biased by the low incidence of cancer pain in the study subjects. Third, other potential confounders such as cancer prognosis (stage, performance status, and QOL), type of treatment and smoking-related information (the number of cigarettes smoked per day, nicotine dependence score) should ideally have been considered. However, all study subjects were in a good condition, i.e. they were well enough to receive their routine annual health checkup as employees at the time of cancer diagnosis. We attempted to minimize the effect of this confounding in multivariable analysis. Also, influence of passive smoking on the strong opioid use was not considered in this study.

## Conclusions

Our study demonstrated that continuing to smoke after cancer diagnosis is associated with later severe cancer pain. Importantly, those patients who quit smoking after cancer diagnosis had a lower risk of severe cancer pain than patients who continued to smoke. Our results contribute one of the evidence that smoking cessation after cancer diagnosis benefits cancer patients. This information provides an evidence base for oncologists and medical staff to strongly encourage their cancer patients to stop smoking.

## Supporting information

S1 Fig Definition of Pattern 1 and Pattern 2 in cases where the duration between cancer diagnosis and cancer pain diagnosis was less than 12 months in the sensitivity analysis. (DOCX)

S2 Fig Sensitivity analyses for patients with less than one year between cancer diagnosis and cancer pain diagnosis. (DOCX)

S1 Table Sensitivity analysis: Factors associated with strong opioid use. (DOCX)
