# Supplementary material for: Smoking cessation after cancer diagnosis reduces the risk of severe cancer pain: A longitudinal cohort study
Source: PLoS One. 2022 Aug 9;17(8):e0272779. doi: 10.1371/journal.pone.0272779 (PMC9362951; doi:10.1371/journal.pone.0272779)
Supplement: S1 Fig — (DOCX) [file pone.0272779.s001.docx]

S1 Fig. Definition of Pattern 1 and Pattern 2 in cases where the duration between cancer diagnosis and cancer pain diagnosis was less than 12 months in the sensitivity analysis.

In pattern 1:

Re-classification from “non-smoker” to “abstainer” for the subjects who had the following history:

annual health check-up

Cancer diagnosis

Stop smoking

Cancer pain diagnosis

(non-smoking)

In pattern 2:

“Current smokers” who did not receive annual health check-ups between cancer diagnosis and cancer pain diagnosis were excluded
because they might include the following “misclassified” cases:

annual health check-up

Cancer diagnosis

Stop smoking

Cancer pain diagnosis

(current smoking)

Smoker
