# Supplementary material for: Smoking cessation after cancer diagnosis reduces the risk of severe cancer pain: A longitudinal cohort study
Source: PLoS One. 2022 Aug 9;17(8):e0272779. doi: 10.1371/journal.pone.0272779 (PMC9362951; doi:10.1371/journal.pone.0272779)
Supplement: S2 Fig — (DOCX) [file pone.0272779.s002.docx]

S2 Fig. Sensitivity analyses for patients with less than one year between cancer diagnosis and cancer pain diagnosis.

.

.

.

.

0

200

400

600

800

1000

analysis time

Non-smoker (n=383)

Abstainer (n=75)

Current smoker (n=84)

Log-rank test: non-smoker group/ current smoker group: p=0.842

non-smoker group/ abstainer group: p< 0.001

　　　　　　abstainer group/ current smoker group: p=0.001

Pattern 2

1.00

Pattern 1

.

0

200

400

600

800

1000

analysis time

Non-smoker (n=188)

Abstainer (n=270)

Current smoker (n=133)

0.00

0.25

0.50

0.75

1.00

0.75

0.50

Proportion of those who do not use strong opioids

Proportion of those who do not use strong opioids

Log-rank test: non-smoker group/ current smoker group: p=0.006

non-smoker group/ abstainer group: p< 0.001

　　　　　　abstainer group/ current smoker group: p<0.001

0.25

0.00

.

.

.

0

200

400

600

800

1000

analysis time

Non-smoker (n=188)

Abstainer (n=54)

Current smoker (n=66)

Log-rank test: non-smoker group/ current smoker group: p=0.650

non-smoker group/ abstainer group: p< 0.001

　　　　　　abstainer group/ current smoker group: p<0.001

Pattern 1: Patients with less than one year between cancer diagnosis and cancer pain diagnosis, and whose smoking status was “not smoking” are judged to be abstainers (n=591).

Pattern 2: Current smokers with less than one year between cancer diagnosis and cancer pain diagnosis who received a health checkup before cancer diagnosis are excluded (n=542).

Pattern 3: All patients with less than one year between cancer diagnosis and cancer pain diagnosis are excluded (n=308).

1.00

Pattern 3

0.75

0.50

Proportion of those who do not use strong opioids

0.25

0.00
