# Supplementary material for: Smoking cessation after cancer diagnosis reduces the risk of severe cancer pain: A longitudinal cohort study
Source: PLoS One. 2022 Aug 9;17(8):e0272779. doi: 10.1371/journal.pone.0272779 (PMC9362951; doi:10.1371/journal.pone.0272779)
Supplement: S1 Table — (DOCX) [file pone.0272779.s003.docx]

|  |  | Pattern 1 (n=591) | | | Pattern 2 (n=542) | | | Pattern 3 (n=308) | | |
| --- | --- | --- | --- | --- | --- | --- | --- | --- | --- | --- |
|  |  | HR | p-value | ［95% CI］ | HR | p-value | ［95% CI］ | HR | p-value | ［95% CI］ |
| Smoking status | Non-smoker | ref |  |  | ref |  |  | ref |  |  |
|  | Abstainer | 1.09 | 0.693 | [0.70-1.69] | 0.89 | 0.678 | [0.53-1.52] | 0.88 | 0.693 | [0.47-1.66] |
|  | Current smoker | 1.94 | 0.004 | [1.24-3.03] | 1.59 | 0.032 | [1.04-2.43] | 2.41 | <0.001 | [1.48-3.93] |
| Age | <60 | ref |  |  | ref |  |  | ref |  |  |
|  | >60 | 0.70 | 0.132 | [0.45-1.11] | 0.67 | 0.109 | [0.41-1.09] | 0.72 | 0.240 | [0.42-1.25] |
| Gender | Female | ref |  |  | ref |  |  | ref |  |  |
|  | Male | 2.11 | <0.001 | [1.49-2.99] | 2.05 | <0.001 | [1.44-2.90] | 2.00 | 0.001 | [1.32-3.04] |
| Alcohol consumption | none/sometimes | ref |  |  | ref |  |  | ref |  |  |
|  | Every day | 0.88 | 0.501 | [0.59-1.29] | 0.87 | 0.528 | [0.57-1.34] | 0.85 | 0.534 | [0.50-1.43] |
| BMI at the time of cancer pain | <18.5 | ref |  |  | ref |  |  | ref |  |  |
|  | ≥18.5 < 25 | 0.75 | 0.260 | [0.45-1.24] | 0.69 | 0.168 | [0.40-1.17] | 0.57 | 0.093 | [0.29-1.10] |
|  | ≥25 | 0.56 | 0.058 | [0.30-1.02] | 0.47 | 0.022 | [0.25-0.90] | 0.24 | 0.001 | [0.10-0.55] |
| Cancer type ^a^ | Not tobacco-related cancer | ref |  |  | ref |  |  | ref |  |  |
|  | Tobacco-related cancer | 1.08 | 0.655 | [0.77-1.51] | 1.12 | 0.541 | [0.78-1.60] | 0.98 | 0.933 | [0.63-1.51] |
| Time since cancer diagnosis ^b^ | (continuous variable) | 1.01 | 0.001 | [1.004-1.017] | 1.01 | <0.001 | [1.005-1.018] | 1.01 | 0.008 | [1.003-1.022] |

S1 Table. Sensitivity analysis: factors associated with the use of strong opioids

a: Tobacco-related cancer: lung, head and neck (except thyroid), esophageal, pancreas, kidney, urinary bladder, renal pelvis, stomach, liver, uterine cervix cancer 　　b: months
